# Microbes without borders: uniting societies for climate action

**DOI:** 10.1093/femsec/fiaf084

**Published:** 2025-09-23

**Authors:** J T Lennon, L S Bittleston, Q Chen, V S Cooper, J Fernández, J A Gilbert, M M Häggblom, L V Harper, J K Jansson, N Jiao, E M Kuurstra, R S Peixoto, R Rappuoli, M A Schembri, A Ventosa, D L Vullo, C Zhang, N K Nguyen

**Affiliations:** Department of Biology, Indiana University, Bloomington, IN, United States; American Academy for Microbiology, Washington, DC, United States; American Society for Microbiology, Washington, DC, United States; American Society for Microbiology, Washington, DC, United States; Department of Biological Sciences, Boise State University, Boise, ID, United States; Innovation Research Center for Carbon Neutralization, Fujian Key Laboratory of Marine Carbon Sequestration, Xiamen University, Xiamen, China; Global Ocean Negative Carbon Emissions Program, Xiamen, China; American Society for Microbiology, Washington, DC, United States; Department of Microbiology & Molecular Genetics, University of Pittsburgh School of Medicine, Pittsburgh, PA, United States; Instituto de Biotecnología y Biología Molecular, Departamento de Ciencias Biológicas, Facultad de Ciencias Exactas, Universidad Nacional de La Plata, CCT La Plata-CONICET, La Plata, Buenos Aires, Argentina; Sociedad Argentina de Investigaciones en Bioquímica y Biología Molecular, Buenos Aires, Argentina; Department of Pediatrics and Scripps Institution of Oceanography, University of California San Diego, La Jolla, CA, United States; Applied Microbiology International, Cambridge, United Kingdom; Department of Biochemistry and Microbiology, Rutgers University, New Brunswick, NJ, United States; Federation of European Microbiological Societies, Delft, The Netherlands; Applied Microbiology International, Cambridge, United Kingdom; Pacific Northwest National Laboratory, Richland, WA, United States; The Soil Stars, Applied Microbiology International, Cambridge, United Kingdom; Innovation Research Center for Carbon Neutralization, Fujian Key Laboratory of Marine Carbon Sequestration, Xiamen University, Xiamen, China; Global Ocean Negative Carbon Emissions Program, Xiamen, China; Federation of European Microbiological Societies, Delft, The Netherlands; Division of Biological and Environmental Science and Engineering (BESE), King Abdullah University of Science and Technology (KAUST), Thuwal, Saudi Arabia; International Society of Microbial Ecology, Wageningen, The Netherlands; Fondazione Biotecnopolo di Siena, Siena, Italy; International Union of Microbiological Societies, Utrecht, The Netherlands; Institute for Molecular Bioscience, and School of Chemistry & Molecular Biosciences, The University of Queensland, Brisbane, Queensland, Australia; Australian Society for Microbiology, Fitzroy, Victoria, Australia; Federation of European Microbiological Societies, Delft, The Netherlands; Department of Microbiology and Parasitology, University of Seville, Seville, Spain; Environmental Biotechnology Lab, Área Química, Instituto de Ciencias, Universidad Nacional General Sarmiento-CONICET, Buenos Aires, Argentina; Sociedad Argentina de Microbiología General, Buenos Aires, Argentina; Global Ocean Negative Carbon Emissions Program, Xiamen, China; Shenzhen Key Laboratory of Geo-Omics of Archaea, Southern University of Science and Technology, Shenzhen, China; American Academy for Microbiology, Washington, DC, United States; American Society for Microbiology, Washington, DC, United States

The climate crisis is one of the most urgent and complex challenges of our time. Although often overlooked in models and policy, microorganisms play a critical role in climate dynamics. They are sensitive to environmental drivers such as rising temperatures and altered precipitation patterns, with far-reaching consequences for the health of crops, livestock, and human populations. Climate change can also disrupt biogeochemical cycles that microbes help regulate, thereby altering feedbacks that influence Earth system processes (Lennon et al. 2024). Yet, microbes offer powerful and unique opportunities for climate change mitigation. In both natural and industrial contexts, microbial life can be leveraged to reduce emissions (Jiao et al. 2024), restore ecosystems, and enhance resilience (Tiedje et al. 2022, Rappuoli et al. 2025). Realizing this potential will require coordinated action and shared goals across societies, stakeholders, sectors, and borders (Peixoto et al. 2024).

In May 2025, representatives from microbiology societies around the world convened in Washington, DC, for the Global Strategy Meeting on Microbes and Climate Change, hosted by the American Society for Microbiology (ASM). This inaugural gathering marked the launch of a global alliance to establish microbial science as a pillar of climate action. The meeting brought together leaders from North America, South America, Europe, Asia, Australia, and the United Kingdom, representing diverse organizations, including Applied Microbiology International (AMI), the Sociedad Argentina de Investigaciones en Bioquímica y Biología Molecular (SAIB), the Australian Society for Microbiology (which also uses the acronym ASM), the Federation of European Microbiological Societies (FEMS), the International Society for Microbial Ecology (ISME), the International Union of Microbiological Societies (IUMS), the Sociedad Argentina de Microbiología General (SAMIGE), Global ONCE, the Soil Stars initiative, and ASM itself.

The meeting identified four major priorities to guide collective action. First, a formal coalition is needed to establish an organized and unified voice for microbial science in climate discourse. A coordinated alliance will enhance credibility, expand influence, and attract funding, while signaling that the global microbiology community is stepping forward with purpose, commitment, and urgency.

Second, microbial science must be embedded in the strategic frameworks that shape climate action and advocacy. Partners like One Earth, who participated in the meeting, are actively seeking microbiology-informed insights to address critical knowledge gaps in existing climate solutions. By engaging with policymakers, funders, entrepreneurs, and advocacy groups, the microbial science community can help align models, policies, and investments with the essential roles that microbes play in global climate systems.

Third, communication of microbial science must be reimagined. Traditional forms of scientific communication are insufficient to influence public understanding or policy discourse around climate change. Scientific societies must invest in more effective communication strategies that prioritize storytelling, advocacy, and media engagement. By partnering with professional communicators, developing accessible and engaging content, and launching global outreach campaigns, the microbial science community can shift microbes from invisible to indispensable in the climate conversation.

Fourth, real-world demonstration projects are essential to showcase the tangible benefits of microbial solutions, such as enhancing coastal carbon sequestration through wastewater treatment or restoring degraded soils with sustainable biofertilizers (3). These efforts can deliver measurable ecological and economic outcomes, foster local engagement and trust, and attract interest from funders, policymakers, and industry leaders. By highlighting the impact of microbial interventions at scale, such projects can accelerate adoption, inform policy to promote government incentives, and generate momentum for broader climate action.

The climate crisis demands a united response. The strategy meeting in Washington, DC, was a first step toward building a global alliance for microbial climate solutions. We invite all microbiology societies and stakeholders to join this initiative. The priorities are clear. Now, we must turn the shared vision into sustained action. Forming the coalition, securing visibility in climate policy, and demonstrating microbial solutions in the real world will require persistence, coordination, and leadership. Microbial life knows no borders. Neither should the effort to harness its potential. The time to come together is now.
